# Molecular Basis of Cardiac Myxomas

**DOI:** 10.3390/ijms15011315

**Published:** 2014-01-20

**Authors:** Pooja Singhal, Adriana Luk, Vivek Rao, Jagdish Butany

**Affiliations:** 1 Department of Pathology, Toronto General Hospital, University Health Network, Toronto, ON M5G2C4, Canada; E-Mail: pooja.singhal@mail.utoronto.ca; 2 Division of Cardiology, Department of Medicine, University of Toronto, Toronto, ON M5G2C4, Canada; E-Mail: adriana.luk@mail.utoronto.ca; 3 Division of Experimental Therapeutics, Cardiovascular Toronto General Research Institute, Toronto General Hospital, Toronto, ON M5G2C4, Canada; E-Mail: vivek.rao@uhn.ca

**Keywords:** primary cardiac tumors, cardiac myxomas, molecular genetics, Carney complex

## Abstract

Cardiac tumors are rare, and of these, primary cardiac tumors are even rarer. Metastatic cardiac tumors are about 100 times more common than the primary tumors. About 90% of primary cardiac tumors are benign, and of these the most common are cardiac myxomas. Approximately 12% of primary cardiac tumors are completely asymptomatic while others present with one or more signs and symptoms of the classical triad of hemodynamic changes due to intracardiac obstruction, embolism and nonspecific constitutional symptoms. Echocardiography is highly sensitive and specific in detecting cardiac tumors. Other helpful investigations are chest X-rays, magnetic resonance imaging and computerized tomography scan. Surgical excision is the treatment of choice for primary cardiac tumors and is usually associated with a good prognosis. This review article will focus on the general features of benign cardiac tumors with an emphasis on cardiac myxomas and their molecular basis.

## 1. Introduction

Cardiac tumors are rare and are divided into primary and metastatic tumors with an autopsy series showing metastatic cardiac tumors to be about 100 times more common than primary cardiac tumors [1]. In another autopsy series, the prevalence of primary cardiac tumors was found to be 0.001%–0.03% [2]. Amongst primary cardiac tumors, about 90% are benign and of these the most common are cardiac myxomas (50%–80%) [3]. Other benign primary cardiac tumors include papillary fibroelastoma (PFE) (26%), fibromas (6%), lipomas (4%), rhabdomyomas, hemangiomas and atrioventricular node tumor [4]. Recent studies show that papillary fibroelastomas are the most common primary benign cardiac tumors [5,6]. Amongst the primary malignant cardiac tumors, the most common ones are sarcomas (90%) followed by lymphomas [4].

### Clinical Features

The clinical presentation of primary cardiac tumors depends on their site, size, mobility and infiltration of adjacent structures. Up to 12% of primary cardiac tumors are completely asymptomatic and are diagnosed at incidental investigation (most commonly an echocardiogram) or at postmortem examination [7]. Others present with one or more signs and symptoms of the classical triad of hemodynamic changes caused by a mobile intracardiac mass, with obstruction, pulmonary or systemic embolism and nonspecific constitutional symptoms [7,8]. Malignant primary cardiac tumors may cause symptoms relatable to metastases to different organs such as the lungs, brain and bones.

## 2. Cardiac Myxomas

Cardiac myxomas (CM) are the most common benign primary cardiac tumors in adults with an incidence of 0.5–1 case per 10^6^ individuals per year [9]. They are three times more common in females and 90% are diagnosed in the fourth to sixth decades of life [6,10]. They are rarely seen in children, in whom they constitute 15% of cardiac tumors [6,11,12].

CM can be seen anywhere in the heart, but arise most commonly in the left atrium (60%–80%), in the region of the interatrial septum and the fossa ovalis [6,8], followed by the right atrium (15%–28%), the right ventricle (8%) and the left ventricle (3%–4%) [8,11]. About 10% of CM are reportedly biatrial, however, this may be a misconception, since a close examination of these, has shown that the neoplasm extends through the fossa ovalis, usually from the left to the right side [13]. Ventricular myxomas are usually seen in women and children [14]. Occasional case reports of CM originating from the mitral and aortic valves, pulmonary vessels, inferior vena cava and superior part of the interventricular septum do appear in literature [15–18].

About 90% of CM occur sporadically, while 5%–10% of cases show a familial inheritance and occur as a part of the Carney complex (CNC) [19,20]. The Carney complex, first described in 1985 [21], is an X-linked autosomal dominant disorder which shows complete penetrance but variable phenotypic expression. It is characterized by CM, extracardiac myxomas (mucosal and cutaneous), osteochondromyxoma, spotty skin pigmentation, myxomatous tumors of the breast, ductal adenoma of breast, blue nevi, endocrine overactivity and tumors (hypercortisolism, pituitary adenoma with acromegaly or gigantism, thyroid tumors, testicular large cell calcifying Sertoli cell tumors (LCCST) and psammomatous melanotic schwannoma (PMS)) and paradoxical positive response of urinary glucocorticoids to dexamethasone administration (PPNAD) during Liddle’s test [22,23]. The diagnosis of CNC is made in the presence of two or more major manifestations of the syndrome, or in the presence of one major criterion if the patient is a carrier of inactivating mutation of *PRKAR1A* (cyclic AMP-dependent protein kinase type I-alpha regulatory subunit) [24].

Although histologically similar, sporadic myxomas are usually seen in middle-aged women as solitary left atrial masses. Familial myxomas are often multicentric, seen in a younger age group, in sites other than the left atrial septum, show no female predilection, and are prone to recurrence in greater than 20% of cases following surgical resection [7,14,25].

### 2.1. Histogenesis

CM are benign neoplasms whose cell of origin is still not fully established, though they are believed to develop from multipotent mesenchymal stem cells present in the fossa ovalis and surrounding the endocardium [3,14].

Sakamoto *et al*. supported the hypothesis that CM arise from the primitive mesenchymal cells which are capable of undergoing cardiogenic, neuroendocrine and endothelial cell differentiation by demonstrating increased expression of endothelin-1 (*ET-1*), interleukin-6 (*IL-6*), interleukin-8 (*IL8*), chemokine ligand 1 (*CXCL1*) and growth related oncogenes, and absence of stem cell factor, hepatocyte growth factor and granulocyte colony stimulating factor in CM [26]. In addition, some CM also express genes specific for chondrocyte development (sex determining region-box9 (*SOX9*), melanocyte inhibitory activity (*MIA*) and secreted phosphoprotein1 (*SPP1*)), which further support this hypothesis [27].

CM express transcription factors for primitive cardiomyocyte phenotype (Nkx2.5/Csx, GATA-4, MEF2 and eHAND), stem cell markers for endothelial cell precursors (Flt-1 and FlK-1), markers of primitive endothelial cells (CD34), and markers of early cardiogenic differentiation CALB2, MMP2, TIMP-1, Sox9, Notch1 and MMP-1 [28–30]. They also express α-smooth muscle actin (α-SMA), which is expressed in cardiac muscles in the early period of fetal development. They are negative for myosin light chain kinase v2 (MLC-2v) and α-skeletal actin (α-SKA) and focally express α-cardiac actin (α-CA), all of which are markers of terminally differentiated cardiac myocytes, suggesting that CM develop from multipotent mesenchymal progenitors that show cardiomyogenic differentiation [28,30–32]. In addition, coexpression of CD34 and α-actin by some CM further supports their origin from a common cardiac early precursor cell [30].

Pucci *et al*. [33] detected the expression of neuroendocrine markers such as protein gene product 5.5/PP9.5, S100 and neuron-specific enolase (NSE) in 94%, 89% and 57% cases, respectively of CM. In addition, 57% of cases of CM were positive for all these markers, while the chondroid looking areas were positive for S100 and NSE [33]. Teraccianno *et al*. also detected strong and diffuse cytoplasmic and nuclear expression of calretinin (CALB2) in CM, suggesting neuroendocrine differentiation [34].

CM express markers of endothelial cell differentiation such as the von Willebrand factor vWF/FVIII, CD31, CD34 and Ulex europeus agglutinins (UEA-1) in vascular endothelium cells, vascular like aggregates and in stromal cells, suggesting endothelial differentiation [33].

The glandular epithelial structures sometimes seen in CM express epithelial cell markers CK9p and CEA (Carcinoembryonic antigen) and suggest epithelial differentiation [33].

### 2.2. Clinical Presentation

About 10%–15% of patients with CM are asymptomatic at the time of diagnosis [35]. Non-specific constitutional symptoms such as fever, lethargy, physical weakness, fatigue, anorexia, painful erythema, loss of weight and appetite, are seen in up to 90% of cases due to autocrine cytokine production such as IL-6 and IL-8 [8,36]. Non-specific laboratory findings such as chronic hemolytic anemia and thrombocytopenia (due to cellular destruction caused by abnormal blood flow across the tumor surface), polycythemia, erythrocytosis and leukocytosis, raised erythrocyte sedimentation rate, serum *C*-reactive proteins and immunoglobulins may also be seen in some CM [37,38]. These can lead to misdiagnosis of infective endocarditis, rheumatic heart disease, vasculitis, rheumatoid arthritis, and collagen vascular diseases and often resolve after tumor resection [7,39–42].

About 70% of CM can present with signs and symptoms of intracardiac obstruction such as left and right sided heart failure, with dyspnea, orthopnea, paroxysmal nocturnal dyspnea, ascites, hepatomegaly and peripheral edema [43,44]. Large atrial myxomas can cause episodic mitral or tricuspid valvular stenosis and the patient can present with intermittent syncope, dizziness, or sudden death [45]. Left atrial myxomas can produce valvular insufficiency due to the “wrecking ball effect” produced by the back and forth motion of the mass which interferes with valvular closure and can damage the chordae tendinea [46]. Right atrial myxomas can embolize to pulmonary vessels and produce signs of pulmonary hypertension [47]. Large ventricular myxomas can produce signs and symptoms of pulmonary and aortic valvular stenosis [48].

Embolic manifestations are seen in 30%–50% cases of CM, and these are due to fragmentation, detachment and dissemination of parts of the tumor, overlying thrombi or vegetation [38]. The emboli most commonly involve cerebral and retinal arteries (>50%) producing signs and symptoms such as vision disorders, cerebral infarcts, seizures, hemiparesis, aphasia, and progressive dementia [49,50]. Other arteries involved are those of the lower extremities producing claudication, visceral, renal and pulmonary arteries producing pulmonary hypertension and nonspecific symptoms such as myalgia, arthralgia, hyperhidrosis, facial edema and nocturnal hemoptysis [38,51,52]. Very rarely CM may embolize to coronary arteries and can produce myocardial infarction [53,54].

In extremely rare cases, infected CM can present as systemic bacteremia, mycotic embolism, disseminated intravascular coagulation, pulsatile tinnitus, back-ache and hair loss [55,56].

### 2.3. Macroscopy

**Figure 1.**
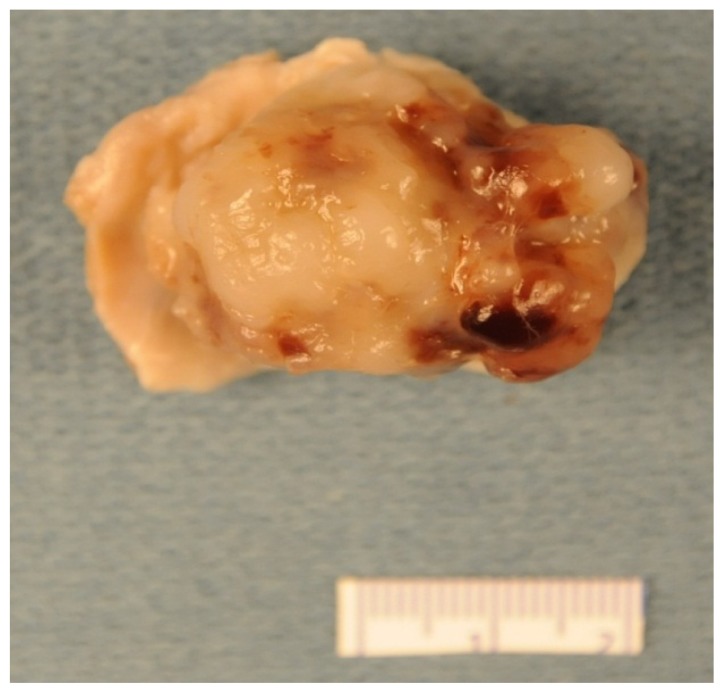
Gross picture of myxoma that is round to oval in shape and has smooth or slightly bosselated surface.

**Figure 2.**
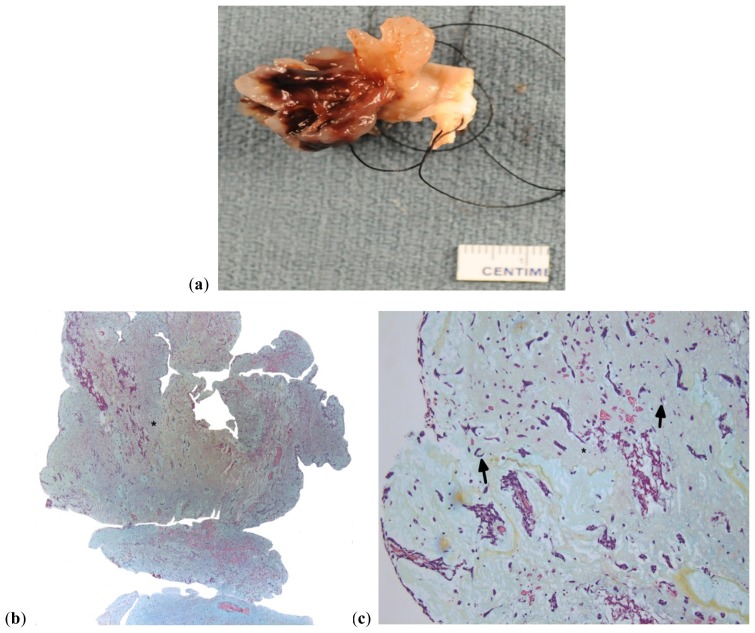
(**a**) Gross picture of a soft myxoma with multiple finger-like projections; (**b**) Histologic section shows a soft friable myxoma (asterisks) with multiple villous projections; (**c**) Higher magnification of the same showing stellate lepidic cells (black arrow) in a myxoid background (asterisks). ((**b**,**c**) Stain: Movat pentachrome; Original magnification (**b**) ×1.2; (**c**) ×10).

Approximately two-thirds of CM are round to oval, sessile, polyploid masses of varying sizes (average 5–6 cm), with a short broad base and smooth or slightly bosselated, glistening surface covered with thrombus and are attached to the interatrial septum (Figure 1). The cut surface is usually bosselated and has a variegated appearance composed of fibrous, gelatinous, myxoid and hemorrhagic areas. One-third of CM are soft, gelatinous, fragile, with papillary or finger-like villous extensions, and prone to fragmentation, embolization and erosion [8,53] Figure 2a–c. The polyploid myxomas usually cause obstructive symptoms while papillary myxomas are usually associated with embolic and neurologic manifestations [53,57].

### 2.4. Histopathology

**Figure 3.**
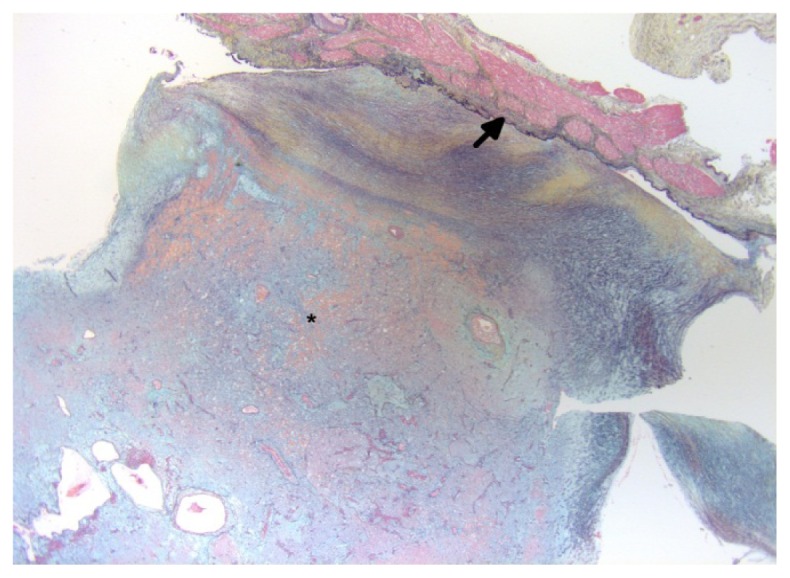
Section shows myxoma (asterisks) arising from endothelial layer (black arrow) (Stain: Movat pentachrome; Original magnification ×1.2).

**Figure 4.**
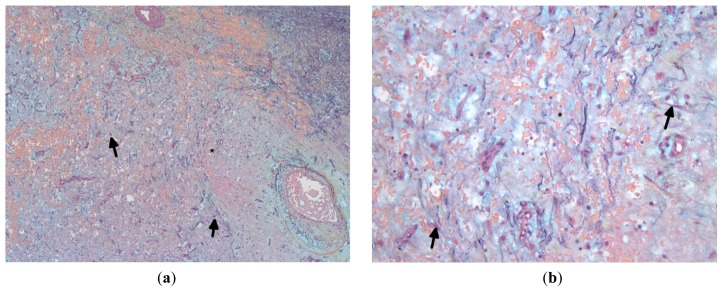
(**a**,**b**) Sections show stellate lepidic cells (black arrow) in a myxoid background (asterisks). ((**a**,**b**): Movat pentachrome; Original magnification: (**a**) ×5; (**b**) ×20).

**Figure 5.**
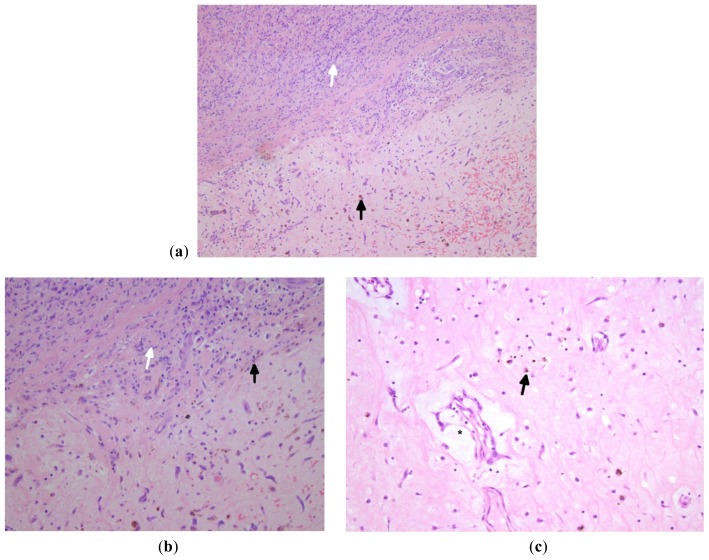
(**a**) Section shows chronic inflammatory cells (lymphocytes and plasma cells (white arrow) and hemosiderin-laden macrophages (black arrow) at the base of the lesion; (**b**) Higher magnification of the same; (**c**) Higher magnification showing hemosiderin-laden macrophages (black arrow) and ring structure (asterisks) ((**a**–**c**): Stain: Hematoxylin and Eosin; Original magnification: (**a**) ×2.5; (**b**) & (**c**) ×20).

**Figure 6.**
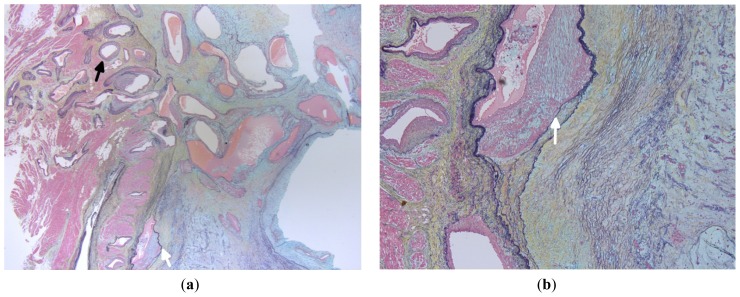
(**a**,**b**) Sections from the base of cardiac myxoma shows increased vascularity with thick walled blood vessels (black arrow) some of which show intimal hyperplasia (white arrow). (Stain (**a**,**b**): Movat pentachrome; Original magnification: (**a**) ×2.5; (**b**) ×5).

**Figure 7.**
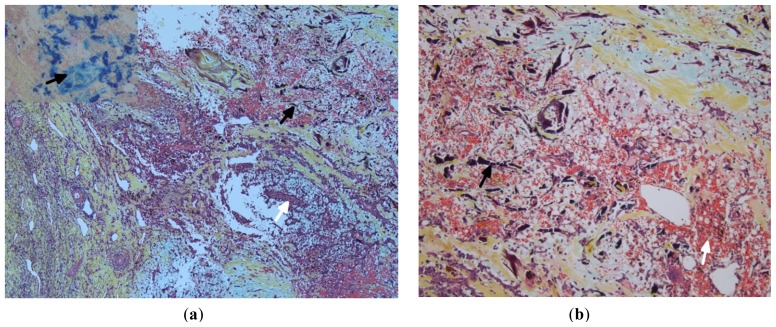
(**a**,**b**) Sections show Gamna-Gandy bodies (black arrow) and areas of fresh and old hemorrhage (white arrow). Inset shows positive iron stain in the Gamna-Gandy bodies. (Stain (**a**,**b**): Movat pentachrome; Original magnification: (**a**) ×5; (**b**) ×10).

**Figure 8.**
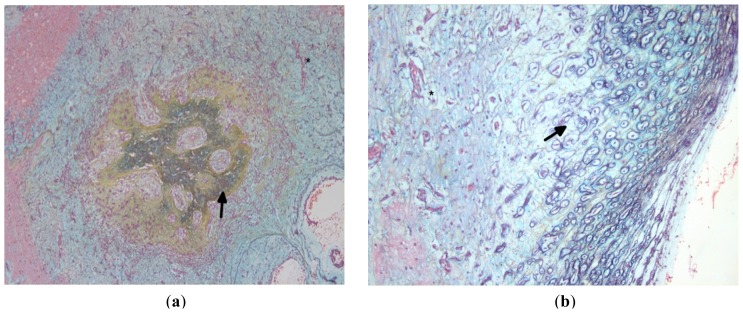
(**a**) Section shows bone (black arrow) in myxoma (asterisks); (**b**) Section shows cartilage (black arrow) in myxoma (asterisk). (Stain (**a**,**b**): Movat pentachrome; Original magnification: (**a**) ×5; (**b**) ×10).

CM arise from the endocardium, do not invade the interatrial septum or underlying myocardium and are covered by a single layer of flat endothelial cells [8,42,58] (Figure 3). They are characterized by the presence of stellate, polygonal (lepidic) cells in an amorphous, basophilic, afibrillar myxoid stroma containing mucopolysaccharides [6,14] (Figure 4a,b). The cells have indistinct cell membranes, a small amount of eosinophilic cytoplasm, round, oval or elongated nucleus and dispersed chromatin. At the surface of the lesion, the cells are arranged in the form of single cells, parallel clusters, syncytial cords, tubular structures or perivascular cuffing [30,59]. Inflammatory cells may be present throughout the lesion. The base of the lesion usually contains prominent chronic inflammatory cells (lymphocytes, plasma cells, and macrophages), areas of old and recent hemorrhage with hemosiderin-laden macrophages, and thick walled vessels, which are likely related to the solid phenotype and chronicity of the lesion [13,57,60] (Figures 5a–c, 6a,b). Occasionally, multinucleated tumor giant cells, blood cells, histiocytes, fibroblasts, smooth muscle cells, mucin-forming glands, ring structures (single or multiple concentric layers of myxoma cells surrounding the capillaries), artery-like vessels, superficial thrombi, calcification, chondrocytes, osteoblasts, metaplastic bone, cysts, Gamna-Gandy bodies (degenerated collagen encrusted with iron or calcium), thymic rests and foci of extramedullary hematopoiesis are also seen (Figures 7a,b, 8a,b). Very rarely, mitotic figures can also be seen [6,8,14,61–63].

### 2.5. Immunohistochemistry

**Figure 9.**
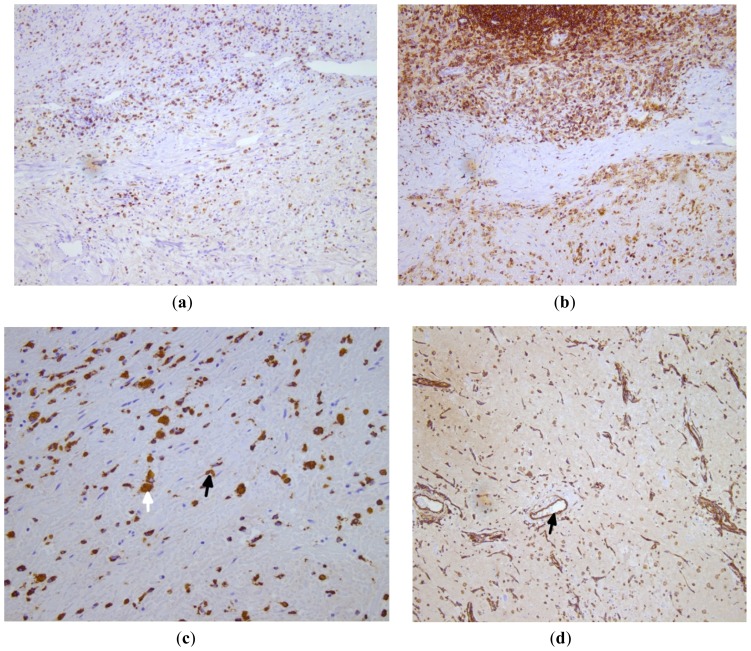
Sections from cardiac myxoma show (**a**) CD8 positive T lymphocytes; (**b**) CD45 positive cells; (**c**) CD68 positive macrophages (black arrow) and hemosiderin-laden macrophages (white arrow); (**d**) CD31 positivity in the blood vessels (black arrow); (Original magnification: (**a**–**c**) ×10; (**d**) ×20).

About 80%–90% of CM express vimentin, Notch1, α smooth muscle actin (α-SMA), calretinin, caldesmon and tenascin C. CD34 positivity is seen in 66.7% cases of CM which stains multinucleated cells in superficial tumor area and lacunae, ring structures, and endothelial cells in arterial-like structures. α-SMA positivity is seen in interstitial and perivascular multinucleated cells and in parietal cells in vascular and ring structures. Other occasionally positive immunohistochemical stains in CM are MMP1, MMP2, TIMP-1 (36.7%), factor VIII, (36.7% in vascular structures), cytokeratin (6.7% in gland like structures), Flt-1 (26.7%), S100 (13.3%) and α-cardiac actin (10%) [30]. Some cases of CM show positive staining for: PGP9.5, NSE, synaptophysin, endothelin-1 in lepidic cells; for CD8, CD45 and CD68 in lymphocytes and hemosiderin-laden macrophages; for UEA and CD31 in vascular structures; and EMA, CEA, NSE, S100 and chromogranin in glandular structures [26,33,62,64] Figure 9a–d.

### 2.6. Electron Microscopy

Electron microscopy of CM shows lepidic cells with single, round, elongated nuclei, with or without nucleolus. The cytoplasm of these cells shows abundant rough and smooth endoplasmic reticulum, polyribosomes, lysosomes, varying shapes and sizes of mitochondria, pinocytic vesicles, numerous filaments and iron deposits [65,66].

### 2.7. Differential Diagnosis

The differential diagnosis of CM includes organizing thrombus, and primary or metastatic sarcomas such as low-grade fibromyxoid sarcoma, myxofibrosarcoma, myxoid liposarcoma, and inflammatory myofibroblastic tumor [58,67,68]. Rare case reports of myxoid leiomyosarcoma and angiosarcoma mimicking clinically, radiologically and grossly as myxoma are present in the literature [69–71]. However, histological features such as presence of “ring structure”, absence of cytologic atypia, mitotic activity, myocardial invasion, metastasis and recurrence (except in Carney complex), slow growth rate and immunohistochemistry in CM helps to differentiate it from primary or metastatic sarcomas (Table 1) [3,67,72–74].

#### 2.7.1. Organizing Thrombus

The perivascular arrangement of myxoma cells helps to differentiate it from organizing thrombus [58].

#### 2.7.2. Inflammatory Myofibroblastic Tumor

Inflammatory myofibroblastic tumor is characterized by the presence of loosely arranged plump spindled myofibroblasts in an edematous myxoid background with abundant blood vessels and an infiltrate of lymphocytes, plasma cells and eosinophils resembling granulation tissue. Occasional mitosis and foci of necrosis may be present. The tumor cells are large with oval vesicular nuclei and prominent nucleoli. Nuclear pleomorphism and mitotic figures are seen [74].

#### 2.7.3. Low-Grade Fibromyxoid Sarcoma (LGFMS)

Low-grade fibromyxoid sarcoma is a variant of fibrosarcoma that is characterized by a mixture of heavily collagenized, hypocellular zones and more cellular myxoid nodules. Bland spindle cells are seen in short fascicles and whorling growth patterns. Tumor cells are small, with poorly defined, pale eosinophilic cytoplasm, round to ovoid nuclei and absent to indistinct nucleoli. The vasculature consists of an arcade of small vessels, and arteriole-sized vessels with perivascular sclerosis. The myocardial infiltration and scattered hyperchromatic cells may be seen but mitosis and atypia are extremely rare. It is differentiated from CM through the presence of myxoma cells, abundant organizing hemorrhage, absence of mitotic figures, and high cellularity in the latter [67,68,75].

#### 2.7.4. Myxoid Liposarcoma (MLS)

Myxoid Liposarcoma is a malignant tumor composed of a mixture of uniform round to oval shaped primitive non-lipogenic mesenchymal cells and small signet ring lipoblasts in a prominent myxoid stroma, rich in a delicate arborizing, “chicken wire” capillary vasculature. Large pools of extracellular mucin and interstitial hemorrhage are seen. The typical MLS lacks nuclear pleomorphism and significant mitotic activity. The presence of lipoblasts and “chicken wire” vasculature and the absence of “ring structures” help to differentiate MLS from CM [67,68].

#### 2.7.5. Myxofibrosarcoma

Myxofibrosarcoma was previously considered a myxoid variant of malignant fibrous histiocytoma (MFH) [76]. The cellular myxofibrosarcoma is easy to differentiate from CM because of the presence of spindle and histiocyte like cells in a herring-bone pattern. However, the hypocellular myxofibrosarcoma is difficult to differentiate from CM. The hypocellular variant is characterized through the presence of plump, spindled or stellate hyperchromatic tumor cells in a myxoid background. A characteristic finding is the presence of prominent, elongated, curvilinear thin walled blood vessels and vacuolated neoplastic fibroblastic cells (pseudo lipoblasts). The tumor cells exhibit minimum nuclear pleomorphism and mitosis [67,68,77].

#### 2.7.6. Others

Leiomyosarcoma is composed of compact bundles of spindle shaped cells with blunt-ended nuclei oriented at sharp angles or 90° to one another in a myxoid background. The tumor cells are arranged in the form of palisade or storiform, and have cytoplasmic glycogen and perinuclear vacuoles. The tumors cells show marked nuclear pleomorphism and mitotic figures. Large areas of necrosis are also seen. Angiosarcomas are characterized by the presence of irregular, anastomosing, sinusoidal vascular channels and papillary structures lined by pleomorphic and atypical cells with large amounts of eosinophilic cytoplasm, hyperchromatic nuclei and prominent eosinophilic nucleoli. The cells show marked nuclear pleomorphism, mitoses and occasional cytoplasmic vacuoles [67,77].

## 3. Diagnosis of Primary Benign Cardiac Tumors

Various radiological investigations are recommended to diagnose cardiac tumors and to differentiate them for other cardiac masses such as vegetations and thrombi (Table 2). Echocardiography is highly sensitive, and specific imaging modality to detect cardiac tumors [78]. It provides excellent anatomical and functional information and is the only imaging modality required preoperatively [72].

## 4. Treatment

The treatment of choice for benign primary cardiac tumors is surgical excision. Immediate surgical excision is indicated in CM and large (>1 cm) PFE because of the high risk of embolism [87,88]. Surgery is also indicated for fibromas, lipomas and lipomatous hypertrophy causing hemodynamic compromise. Palliative tumor debulking is done in large unresectable tumor with rapidly progressive symptoms. Small asymptomatic PFE and rhabdomyomas do not usually require surgical excision because the latter have a tendency to undergo spontaneous regression [79]. Preoperative chemotherapy followed by surgical excision is indicated in some primary malignant cardiac tumors. Primary cardiac lymphomas are treated by systemic chemotherapy with or without radiotherapy [89]. Rarely heart transplantation is indicated in cardiac fibromas and inoperable cardiac tumors.

## 5. Prognosis

Prognosis of primary benign cardiac tumors is excellent following surgical excision [6]. However, intracardiac recurrence after surgical excision is seen in 12%–22% of familial cases of CM and 1%–4% of sporadic cases and occurs due to undiagnosed multicentric primary lesion, incomplete surgical removal and existence and proliferation of reserve cells in the myocardium [14,90]. Extracardiac recurrence of CM occurs due to intraoperative dissemination and survival of neoplastic cells in embolic fragments or overlying thrombi, and their growth at the site of dissemination [3].

## 6. Molecular Genetics of Cardiac Myxomas

There are 34 protein markers reported so far to be involved in the histogenesis and development of CM [38]. (Table 3) These markers have overlapping functions (such as: development of cell, heart, muscle, epithelial, ectoderm, epidermis, skeletal muscle, ossification, bone; cell proliferation, adhesion, migration; endothelial to mesenchymal transformation; and angiogenesis and differentiation to mesenchymal cells, neuronal cell and muscle cells) and act through overlapping signalling pathways (such as G protein coupled receptors, TGFβ receptor, VEGF receptor, MAP kinase, growth receptor signalling pathways, cytokine-cytokine receptor and intracellular signalling cascades) [14,38].

Sporadic CM show upregulation of protein gene product 9.5, S100, neuron specific enolase, CALB2, THBD, calretinin, bFGF, FGFR1, SOX9, NOTCH1 and NFATc1 [14]. Microarray analysis has detected that combined expression of MIA, PLA2G2A and PLTP is highly specific for CM [27].

Papillary myxomas show increased expression of matrix-metalloproteinases such as MMP-1, MMP-2 and MMP-9, which causes degradation of the extracellular matrix and promotes tumor embolization [100]. Increased expression of MUC1, IL6 and α1-globulin in CM is associated with increased risk of embolization [36,101]. Recently plakophilin-2 has been identified as adherens junction protein in CM [104].

### 6.1. Markers of Poor Prognosis

Sporadic CM shows a 10-fold greater expression of MIA (melanoma inhibitory activity) and altered expression of S100 protein, both of which are markers of poor prognosis in malignant melanoma, and correlate with malignant transformation [27,107]. However their exact role in sporadic CM as a marker of malignant transformation needs further evaluation [38]. CM also coexpress CXCL1 (angiogenic factor) and growth related oncogenes, which promote their malignant potential. Overexpression of IL-6, VEGF, PCNA, FGFβ, FGFR1, VEGF, VEGFR1 and VEGFR2 is seen in highly proliferative, angiogenic and malignant myxomas [14].

### 6.2. Drug Targets in CM

The main disease pathways in CM include (CCR2, FMOD-TGFβ, S100-FGFR, NKX2.5-GATA4-SOX9-FGFR, HAND1-GATA4 and MUC1), through which other disease pathways work. The potential drug targets in CM were identified to be the key-nodes (CCR2, TGFβ, MUC1, FGFR, EGFR, GATA4 and HAND1) along with their upstream and downstream regulators (MYC, FOS and MMP9). Blocking the key nodes and their regulators can block the pathways involved in CM development [14,38].

### 6.3. Cytogenetic

DNA analysis has detected that CM have a diploid or aneuploid DNA pattern and exhibit heterogenicity in karyotype [8,35]. Cytogenetic analysis has revealed two major loci of susceptibility genes in CNC [108,109].

The *CNC1* susceptibility gene is the *PPKAR1A* gene, which acts as a tumor suppressor gene. It is found in 80% of cases of familial CNC, is located at 17q22–24, and encodes for R1α subunit of cAMP dependent protein kinase A (PKA), involved in the G protein receptor signalling pathway [13,23]. There can be 80 different mutations in the *PRKAR1A* gene of which the most common leads to a premature stop codon (short premature transcripts) and subsequently nonsense mediated mRNA decay (NMD), which leads to R1α haploinsufficiency (absence or reduction in the mutant protein level) and increased PKA enzyme activity [110,111]. However, occasionally when *PRKAR1A* mutations are not associated with NMD, the outcome of the disease is aggressive [20]. Bertherat *et al*. have identified a hot spot c.491–492del TG mutation which was more significantly associated with CM. They also suggested a role of environmental factors in the pathogenesis of CM considering earlier and frequent occurrence of PPNAD disease in female carriers having PRKAR1A mutation [112].

Recently, missense mutation (Arg674Gln) in another *CNC* gene, perinatal isoform of the myosin heavy chain gene *MYH8*, located at 17p12–31, has been identified. Although the exact mechanism of CM development is not known, it is proposed that mutation in *MYH8* promotes the survival of multipotent progenitor cells in the mature heart and provides a substrate for secondary tumorigenic events [112].

A small subset of families with CNC syndrome shows amplification in the *CNC2* gene, located on 2p16, without *PRKAR1A* mutation. These subjects have negative family history, present later in life and usually do not develop myxomas, PMS, thyroid tumors and LCCSCT [108,110].

In contrast, no single gene mutation has been identified for sporadic CM. However, structural rearrangement in *PRKAR1A* has been identified in one-third of cases of sporadic CM [113].

Experimental models have suggested that various epigenetic factors such as DNA methylation, posttranslational modifications of proteins and miRNA molecules may play a role in the histogenesis of CM by causing reactivation and ectopic expression of early embryonic heart genes such as *NKX2.5/CSX*, *GATA4*, *HOX*, *HAND*, *MYOD*, *SOX4-6*, *S100* and *TGFβ* [14].

It is predicted that the combination of microRNA (let-7, miR-125, miR205, miR-214, miR217 and miR-296) can help in the treatment of CM by targeting the key nodes and their potential upstream and downstream regulators. However, the exact role of these microRNA molecules in the treatment of CM is yet to be established [14].

## 7. Conclusions

CM are amongst the most common primary benign cardiac tumors of uncertain histogenesis. It is postulated that they develop from multipotent mesenchymal stem cells capable of undergoing cardiogenic, neuroendocrine and endothelial cell differentiation. The majority of CM occurs sporadically, while some occur as a part of the Carney complex (CNC). Various studies have detected 34 protein markers that have overlapping functions and act through overlapping pathways and play a role in the development of cardiac myxomas. Cytogenetic analysis has detected two genes, *CNC1* and *CNC2*, which are suspected to be involved in the pathogenesis of the Carney complex. No single gene mutation has been identified for sporadic CM, although, structural rearrangement in *PRKAR1A* has been identified in some cases. The role of epigenetic factors has been suggested in the histogenesis of CM. Various experiments are being conducted to evaluate the role of micro-RNA in the treatment of CM by targeting the key nodes, along with their upstream and downstream regulators. A comprehensive, multi-institutional approach is required to obtain a more complete understanding of the molecular basis and pathogenesis of cardiac myxomas in order to develop improved treatment.

## Figures and Tables

**Table 1.** Immunohistochemical profile of CM imitators.

Tumor	CK	Vim	SMA	Desmin	Myo	S-100	CD31	CD34	FVIII
Inflammatory myofibroblastic tumor	F	+	+	F	−	−	−	−	−
Low grade fibromyxoid sarcoma	−	+	−	−	−	−	−	−	−
Myxoid Liposarcoma	−	−	−	−	−	+	−	−	−
Myxofibrosarcoma	−	+	F	−	−	−	−	−	−
Leiomyosarcoma	F	−	+	+	−	−	−	−	−
Angiosarcoma	F	−	−	−	−	−	+	+	+

**Abbreviations:** CK, cytokeratin; Vim: Vimentin; SMA, α smooth muscle actin; Myo, myogenin; FVIII, Factor VIII; F, Focal; + positive; − negative.

**Table 2.** Investigations in primary cardiac tumors.

Investigations	General considerations	Detects
Chest X-ray	Radiation exposure	Enlarged cardiac silhouette; pericardial effusion; calcification; left atrial enlargement and pulmonary hypertension in CM [8,79]
Transthoracic echocardiography (TTE)	Ideal initial imaging modality, simple, non-invasive, readily available, cost effective	Tumor size, shape, extent, location, attachment, mobility, relationship to adjacent cardiac structures; adherence to the cardiac wall; calcification; hemodynamic consequences [80]
Transesophageal echocardiography (TEE)	Additional resolution than TTE	Better visualization of posterior cardiac segment tumors and small tumors <5 mm; valvular abnormalities (stenosis and regurgitation), adequacy of valvular repair, results of valvular replacement, absence of shunting or leakage around intracardiac patch repair, guides weaning from cardiopulmonary bypass; visualization of left and right atrium and their appendages [6,81,82].
Contrast echocardiography	Contrast nephrotoxicity	Detects tissue perfusion; differentiates tumor from thrombus [83]
Three dimensional echocardiography (3D Echo)		Better temporal and spacial resolution [84]
Cardiac MRI	Expensive, limited availability, no radiation exposure, contrast safer	Staging and treatment planning; best tissue characterization; detects relationship of tumor to adjacent structures; infiltration into the myocardium, pericardium, surrounding structures; tumor vascularity, presence of fat, degree of tissue edema, iron content; incompatible cardiac device; valvular and ventricular function [85]
CT scan	Radiation exposure, contrast nephrotoxicity	Helpful when MRI is contraindicated; staging and treatment planning; better spacial resolution; detects small tumors; tumor vascularity, calcification, presence of fat, thoracic extension; coronary artery assessment [86]
Transvenous cardiac biopsy		Suspected malignancy
Color flow Doppler		Vascularity
CT angiogram		Detects coronary artery disease; involvement of coronary artery by the tumor or by the planned resection

**Table 3.** Molecular markers in cardiac myxomas and their functions.

Functions	Markers
Cell development	EDN1, FGF2, MIB1, NKX2.5, NOTCH1, SPP1,TIMP2
Heart development	ACTC1, EDN1, ENG, GATA4, HAND1, MIB1, MYH10, NFATC1, NKX2.5, NOTCH1, PKP2, SOX9
Epithelial development	CD44, ENG, KDR, MIB1, VEGFA, VEGFR2
Ectodermal and epidermal development	KRT9, NOTCH1, PDGFA, SOX9
Muscle cell differentiation and development	ACTA2, ACTC1, ENG, FGF1, GATA4, HAND1, KRT19, MYH10, NKX2.5, NOTCH1, PDGFRβ, RB1, TNC
Skeletal muscle development	EDN1, FGFR1, MMP2, MMP9, MMP14, PDGFRA, PDGFRβ, PFGF-BB, SPP1, SOX9
Ossification	MMP2, MMP14, SPP1
Bone development	MMP2, MMP14, SOX9, SPP1
Angiogenesis	CD44, EDN1, ENG, FGF2, FGFR1, FLT1, HAND1, IL6, IL8, KDR, MCP1, MIB1, MMP2, MMP14, NKX2.5, NOTCH1, PDGFA, TYMP, VEGFA, VEGFR1, VEGFR2
Extracellular matrix remodeling	MMP1, MMP2, MMP3, MMP9, MMP14
Neural differentiation	CD44, EDN3, FGFR1, IL6, MIB1, MYH10, NKX2.5, NOTCH1, NSE, SPP1, TIMP2, UCHL1,VEGFA
Endothelial to mesenchymal transformation	NFATC1, NOTCH1, SOX9
Mesenchymal cell differentiation	EDN1, EDN3, NFATC1, NOTCH1, SOX9
G protein signaling markers	C3, CCR2, CXCL1,EDN-1, IL8, MCP-1, VIP
Cell proliferation	CXCL1, FGF2, FGFR1, IL-6, IL-8, MIA, MIB1, MMP14, MYH10, NOTCH1,PCNA, PDGF-AA, UCHL1,VEGFA
Cell adhesion	CD34, CD44, CEACAM, ENG, FVIII/vWF, FN1, ITGB4, MCP-1, MIA, MUC5AC, PDGF-AA, PDGFRβ, PFGF-BB, PECAM-1, SOX9, SPP1, TNC, VEGFA
Cell migration and metastasis	CD34,CD44, EDN3, ENG,FGF2, FN1, IL6, IL8, MCP-1, MMP2, MMP9, MMP14, MYH10, PFGF-BB, PDGFRβ, VEGFA, VEGFR1, VEGFR2, VIM
Growth receptor signalling pathway	FOS, MMP9, MYC
VEGFR signaling pathway	PFGF-BB, PDGFRβ
TGFβ receptor signaling pathway	ENG, FMOD, MCP-1, PDGF-AA, SMAD6
MAPK signaling pathway	FGF2, FGFR1, PDGF-AA, PFGF-BB, PDGFRβ, PLA2G2A
Cytokine-cytokine interaction	CXCL-1, CCR2, IL6, IL8, MCP-1, PDGF-AA, PDGFRβ, PFGF-BB, VEGFA,VEGFR1, VEGFR2, VEGFR3
Intracellular signaling cascade	CCR2, CEACAM, CXCL-1, EDN-1,EDN-3, FGF2, IL6, IL8, MCP-1, NFATC1,PCNA, PDGF-AA, RB1,VEGFR1
Enzyme linked receptor signaling pathway	ENG, FGF2, FGFR1, FMOD, MCP-1, PDGF-AA, PDGFRβ, PFGF-BB, SMAD6, VEGFA, VEGFR1, VEGFR2, VEGFR3
Transmembrane receptor serine/threonine kinase signaling pathway	ENG, FMOD, MCP-1, PDGF-AA, SMAD6

**Abbreviations**: ACTA2, actin, alpha2, smooth muscle [33,91]; ACTC1, actin, alpha, cardiac muscle1 [30]; C3, Compliment component 3 [27]; CCR2, chemokine receptor 2 [92]; CD34 and CD44(Indian blood group) [93,94]; CEACAM, carcinoembryonic antigen-related cell adhesion molecule 5 [32,95]; CXCL1, chemokine ligand1 [25]; EDN1 and EDN3, endothelin1 and 3 [26]; ENG, endoglin [27]; FVIII/vWF, factor VIII related antigen/ von Willebrand factor [30,33]; FGF2, fibroblast growth factor2 [96]; FGFR1, fibroblast growth factor receptor1 [96]; FLT1/VEGFR1, fms-related tyrosine kinase 1 (vascular endothelial growth factor receptor) [30,97]; FMOD, fibromodulin [27]; FN1, fibronectin [27]; GATA4, GATA binding protein 4 [28]; HAND1, heart and neural crest derivatives expressed 1 [28]; IL6 and IL8, interleukin 6 & 8 [98]; ITGB4, integrin beta4 [27]; KRT19, keratin19 [63]; MCP1, Monocyte chemoattractant protein1 [92]; MIA, melanocyte inhibitory activity [27]; MIB1, mindbomb homolog1 [99]; MMP, matrix metallopeptidase [100]; MUC5AC, mucin5AC [101]; MYH10, myosin, heavy chain10 [102]; NFATC1, nuclear factor of activated T cells [30]; NKX2-5, transcription factor related locus 5 [28]; NOTCH1, Notch homolog1 [30]; PCNA, proliferating cell nuclear antigen [96,103]; PDGF-AA, alpha platelet derived growth factor alpha [97]; PECAM1, platelet endothelial cell adhesion molecule [33,103]; PKP2, plakophilin 2 [104]; PLA2G2A: phospholipaseA2 [26]; RB1, retinoblastoma1 [105]; SMAD6, family member 6 [30]; SOX9, (sex determining region)-box9 [27,30]; SPP1, secreted phosphoprotein1 [27]; TNC, tenascin C [94]; TIMP2, tissue metallopeptidase inhibitor2 [100]; TYMP, thymidine phosphorylase [92]; UCHL1/PGP9.5, ubiquitin carboxyl-terminal esteraseL1 [33]; VEGFRA, vascular endothelial growth factor A [97,98]; VIM, vimentin [33]; VIP, vasoactive intestinal peptide [106].
